# Future Warming Patterns Linked to Today’s Climate Variability

**DOI:** 10.1038/srep19110

**Published:** 2016-01-11

**Authors:** Aiguo Dai

**Affiliations:** 1 Department of Atmospheric and Environmental Sciences, University at Albany, SUNY, Albany, 1400 Washington Avenue, NY 12222, USA; 2 National Center for Atmospheric Research, Boulder, PO Box 3000, CO 80307, USA

## Abstract

The reliability of model projections of greenhouse gas (GHG)-induced future climate change is often assessed based on models’ ability to simulate the current climate, but there has been little evidence that connects the two. In fact, this practice has been questioned because the GHG-induced future climate change may involve additional physical processes that are not important for the current climate. Here I show that the spatial patterns of the GHG-induced future warming in the 21^st^ century is highly correlated with the patterns of the year-to-year variations of surface air temperature for today’s climate, with areas of larger variations during 1950–1979 having more GHG-induced warming in the 21^st^ century in all climate models. Such a relationship also exists in other climate fields such as atmospheric water vapor, and it is evident in observed temperatures from 1950–2010. The results suggest that many physical processes may work similarly in producing the year-to-year climate variations in the current climate and the GHG-induced long-term changes in the 21^st^ century in models and in the real world. They support the notion that models that simulate present-day climate variability better are likely to make more reliable predictions of future climate change.

Our confidence in climate model predictions of future climate change under a given emissions scenario is largely based on models’ ability to simulate the current mean climate and its variability^1^,^2^,^3^,^4^,^5^. The implicit assumption behind this and most modeling studies^1^,^6^,^7^,^8^ is that the more realistically a climate model simulates the current climate, the more reliable its future predictions may be. The reasoning is that if a model can simulate the present-day processes correctly, it may simulate the climate response to GHG forcing more realistically. However, there has been no direct evidence to support this assumption^1^. To the contrary, it is often assumed that many physical processes underlying the GHG-induced long-term global warming and the year-to-year variability of today’s climate are likely to be different, as the former may involve slower processes, such as the snow/ice albedo feedback and oceanic processes, that work on decadal to centennial time scales and also because of the different nature of the forcings involved^6^,^7^. Although climate models use the same formulations to calculate both the year-to-year variations and GHG-induced long-term changes in surface air temperature and other fields, increases in atmospheric GHGs provide an external long-term forcing to which the climate may respond differently compared with its short-term natural variations resulting mainly from random perturbations such as those generated by midlatitude fronts and tropical cyclones, or short-term external forcing such as volcanic eruptions. Because of this, physical relationships found at the inter-annual time scales may not be relevant for GHG-induced global warming. This has major implications in climate research since our instrumental records and satellite observations of the climate are often too short for examining relationships on decadal to centennial time scales.

Here I provide evidence that suggests **a**) similar physical processes are likely responsible for a large portion of both the short-term variations in today’s climate and the GHG-induced long-term climate changes in the 21^st^ century and **b**) models that simulate current climate variability better may indeed produce more realistic projections of future climate change patterns. Thus, the results provide much-needed evidence for the widely-held assumption and help resolve the above-mentioned contradiction, besides other important implications for climate modeling, evaluation, and prediction.

## Results

**Figure 1:**
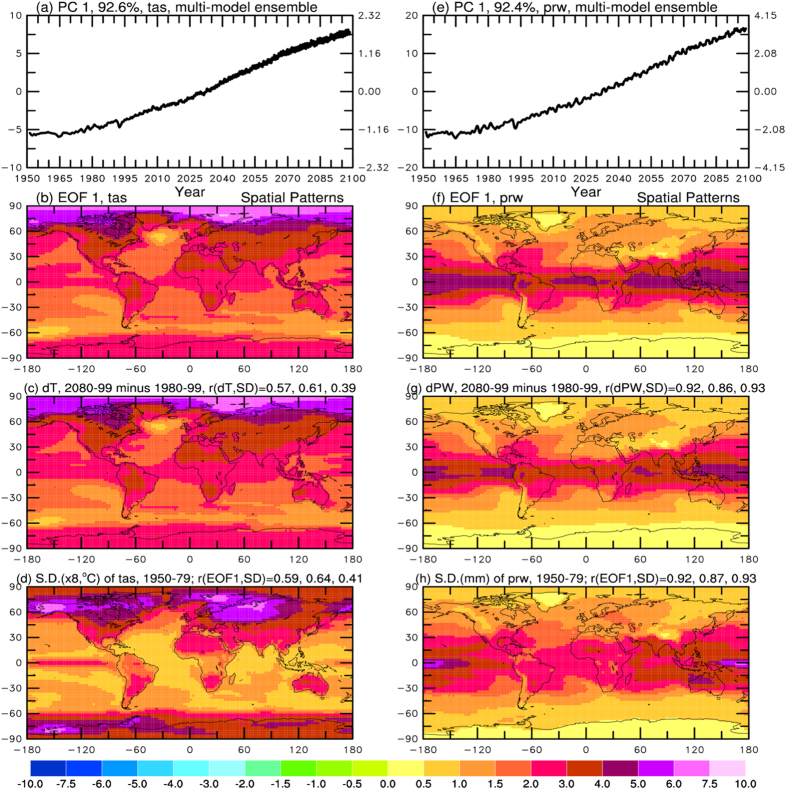
Left column: The principal component (PC, (**a**) left ordinate) and empirical orthogonal function (EOF, (**b**) of the leading mode of the monthly anomalies (relative to the 1950–1979 mean annual cycle, all months combined) of surface air temperature from 22 CMIP3 models from their 20^th^ and 21^st^ (under the A1B scenario) century simulations. The EOF 1 pattern is compared with the patterns in (**c**) 2080–2099 minus 1980–1999 surface air temperature differences (dT, multiplied by 0.9) and (**d**) the standard deviation (S.D., multiplied by 8 and in ^o^C) of the monthly temperature anomalies (all months combined) during 1950–1979, with the pattern correlation coefficient (*r*) between the dT and SD (EOF1 and SD) is shown on top of panel **(c,d)** for, from left to right, all areas, land and ocean only. In (**a**), the global-mean temperature anomalies (^o^C) associated with this EOF is shown on the right-side ordinate, and % variance explained by the mode is given on top of the panel. **Right column:** same as the left column but for precipitable water (PW) from 20 CMIP3 models and the unit is millimeters. To use the same color scale, values in (**f**,**h**) were multiplied by 0.4 and 4.0, respectively. The product of the PC and EOF coefficients yields the anomalies in one tenth of the given unit associated with the mode for a given time and location. Detrending the 1950–1979 data had little effect on the S.D., which includes variations on time scales from a month to 30 years (but with the mean annual cycle excluded). All maps in this paper were created using NCAR Graphics library by the author.

To separate the GHG-induced warming signal from other variations, I performed an empirical orthogonal function (EOF) analysis^9^ of the monthly anomalies of surface air temperature (SAT) and total water vapor content or precipitable water (PW) in an air column for 1950–2099 simulated by and averaged over all the climate models participated in the IPCC Fourth Assessment (AR4)^6^,^10^ under the IPCC A1B scenario for future emissions (a similar analysis was also done using the CMIP5 model data^11^). The first EOF (Fig. 1a-c,e-f) clearly represents the global warming signal with the familiar temporal and spatial characteristics that resemble the global-mean GHG forcing series and long-term change patterns^6^,^7^. Surprisingly, the EOF and long-term change patterns Fig. 1c,g for both the SAT and PW broadly resemble the spatial patterns of the standard deviation (SD) of the monthly anomalies of surface air temperature and PW, respectively, from the models *during 1950–1979* when the warming trend is relatively small (thus detrending had little effect; Fig. 1). This is especially true for PW, whose future change patterns strongly correlate with the SD patterns (r = 0.86–0.93). Besides the obvious land-ocean contrasts seen in both the future change patterns and the current SD maps, the pattern correlation over land or ocean only is also strong, although not perfect. For example, the correlation coefficient between the future PW EOF change patterns and its current SD patterns is 0.87 and 0.93 over land and ocean, respectively; while these numbers are lower but still statistically significant at 0.64 and 0.41 for the SAT case. Although the pattern correlation for the SAT case is not very strong, especially over ocean, the similarities between the future change patterns and current variability patterns are remarkable considering that they are caused by totally different forcing and they result from very different physical processes (e.g., ENSO or volcano induced variations vs. GHG-induced changes).

The EOF represents the mean patterns averaged over the entire data period. To check whether the EOF patterns change over time, I performed the EOF analysis for three different periods from 1950–1999, 2000–2049 and 2050–2099. The results (Fig. S1) show only small changes in the EOF patterns that may be partly due to sampling errors for the relatively short periods, and they are similar to the EOF patterns for the whole period from 1950–2099 (Fig. 1). The pattern correlation with the SD of 1950–1979 is still significant, ranging from 0.85–0.93 for PW and 0.30–0.71 for SAT. The correlation between the SD patterns and future change patterns also exist for each of the seasons (Fig. S2). These results show that change patterns for PW in the 21^st^ century largely resemble the spatial patterns of their variability during 1950–1979, and this is also true for SAT to a lesser degree.

**Figure 2:**
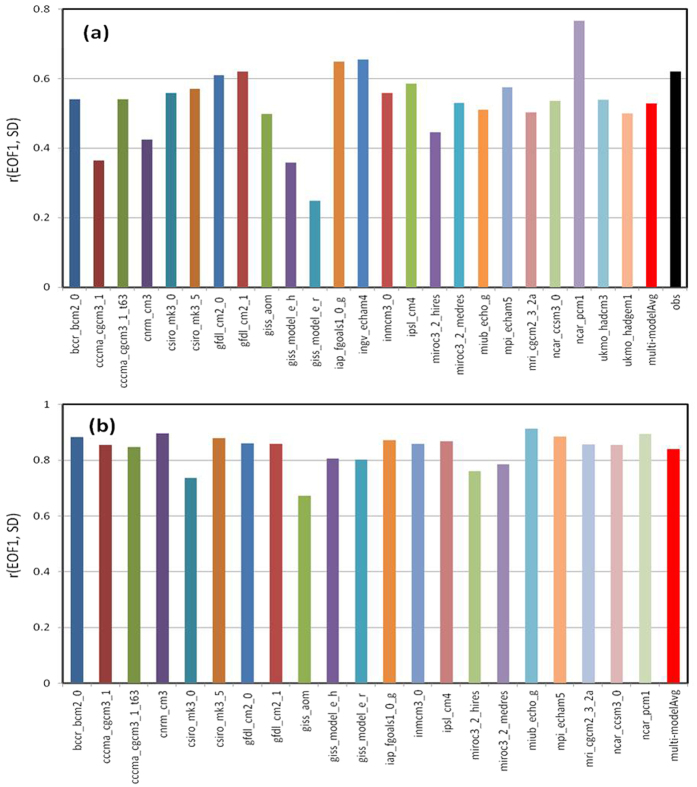
The correlation coefficient between the spatial patterns of the standard deviation of the monthly anomalies during 1950–1979 and the leading EOF of the monthly anomalies during 1950–2099 under the A1B scenario simulated by individual CMIP3 coupled models and the multi-model averaged correlation (red bar) for (**a**) surface air temperature and (**b**) precipitable water. Also shown in (**a**) (black bar) is the case for observed surface monthly temperatures during 1950–2010.

The multi-model ensemble averaging used in Fig. 1 and S1 reduces natural variations and enhances the GHG-induced warming signal, increasing the percentage variance explained by the first EOF from around 35–50% in individual models (not shown) to around 92% (Fig. 1) for 1950–2099. Without the ensemble averaging, the warming signal (EOF1) in the individual models is not separated from other modes of variability as well as for the multi-model ensemble mean case. Nevertheless, the warming patterns, as represented by the leading EOF, of the SAT and PW in the individual models (from one single run) for 1950–2099 are still strongly correlated with the spatial patterns of the SD of the SAT and PW monthly anomalies from the same model during 1950–1979, with most of the models showing a correlation around or above 0.8 for PW and 0.5–0.6 for SAT (Fig. 2). Thus, there exists a significant correlation between the future change patterns of SAT and PW and the patterns of their present-day variations in all the IPCC AR4 models, and this correlation is very strong (~0.8 or higher) for PW in most of the models. I also found significant correlations between the patterns of 1950–1979 SD and the 2080–2099 minus 1980–1999 difference maps for SAT and PW for individual models, although this correlation is slightly lower than those shown in Fig. 2 due to contamination of other modes of internal variability in the simple difference maps, i.e., the 20-year difference maps from individual model runs contain varying contributions from internal decadal variability^12^, which may not be a major contributor for the SD pattern during 1950–1979 in many models.

I also analyzed the CMIP5 model output and found similar pattern correlations (slightly higher for SAT) between future changes and current variability (Fig. S3).

**Figure 3:**
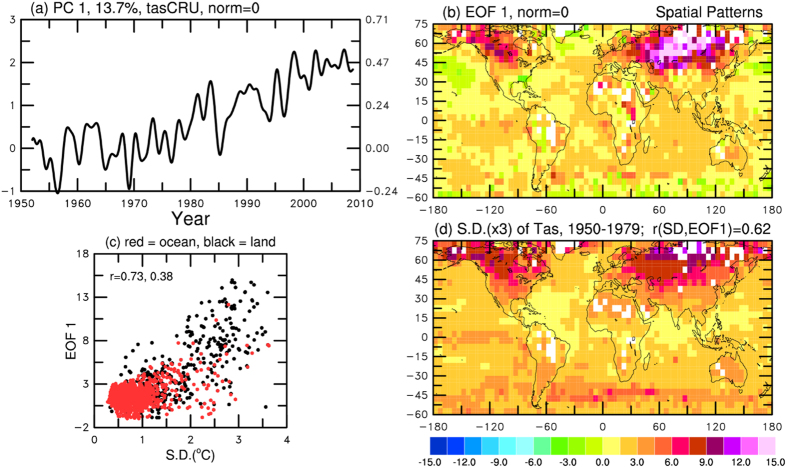
The temporal (**a**) and spatial (**b**) patterns of the leading mode of the monthly anomalies (relative to the 1950–1979 mean annual cycle) of observed surface air (over land) and sea (over ocean) temperature from 1950–2010 (from the Climate Research Unit). (**d**) The standard deviation of the monthly anomalies (multiplied by 3, in ^o^C) during 1950–1979 is shown. (**c**) The scatter plot of the data points (within 60^o^S–75^o^N, land points in black and ocean points in red) shown in (**b**,**d**). The correlation coefficient for all areas, land and ocean are 0.62, 0.73 and 0.38, respectively. Variations on time scales shorter than 24 months were filtered out in (**a**). All maps were created using NCAR Graphics library by the author.

To check whether the above relationship found in models exists in the real world, I performed a similar EOF analysis of observed surface air temperature over land and sea surface temperature over ocean using the Climate Research Unit data set (HadCRUT3)^13^,^14^. I focused on the 1950–2010 period as observations for earlier years are sparse over oceans and many land areas. Data for the polar regions are sparse and thus these regions were excluded from the analysis. Compared with the EOFs from the models, the leading EOF from this data set (Fig. 3a,b) exhibits more short-term variations and more spatial noise. Nevertheless, it still represents a warming trend over most of the globe, especially over land and the Indian and Atlantic Ocean. Although the noisy EOF patterns (Fig. 3b) do not match the SD of the 1950–1979 from the same data set over the North Pacific and several other regions, they are still significantly correlated (*r* = 0.62) for all areas within 45^o^S–75^o^N, which is comparable to those shown in Fig. 2a.

## Discussion

The reason for the similarity between the current variability patterns and future climate change patterns likely lies in the similarity in their underlying physical processes. For both temperature and water vapor content, it is known^15^ that they are less variable (or more stable) under solar heating or other forcing over ocean than over land due to many basic processes such as **a**) higher heat capacity or inertia for water than for land surface, **b**) mixing within the ocean, and **c**) evaporative cooling over water surface. Over land, spatial variations in the variability could arise from heterogeneity in land surface (e.g., surface albedo, soil moisture content, vegetation cover, etc.), while spatial differences in air-sea interactions and oceanic processes can lead to the SD variations over ocean. These basic processes likely also are at work for the GHG-induced climate change case, thus contributing to the similar change patterns. Other physical processes behind the land-ocean warming contrast^16^,^17^, the Arctic warming amplification^18^,^19^,^20^,^21^, and ocean warming patterns^22^,^23^ may also be at work in producing the variability in today’s climate, as there is no reason to expect them not to work in today’s climate. Our results suggest that these processes may work similarly on inter-annual to centennial time scales and respond similarly to short-term perturbations and long-term GHG forcing, and that any additional processes for the GHG-induced climate change do not play a dominant role in determining the global surface warming patterns and other change patterns by the end of the 21^st^ century. This is especially true for PW, which is known to be controlled primarily by atmospheric thermodynamics that links it to air temperature^15^,^24^. This finding implies that models need to be able to realistically simulate the current variability patterns and the underlying processes (e.g., those associated with ENSO) in order for them to realistically simulate future climate change patterns. My results provide a scientific basis for ranking future model projections based on their ability in simulating the current climate, a common practice used in climate research that has been based on common sense rather than scientific evidence. My findings can also be taken as a cautionary message, and they support the notion that models with large errors in simulating the spatial patterns associated with ENSO and other variability are likely to perform poorly in predicting future climate change patterns.

The above analysis compares the spatial patterns only; it does not address the magnitude of change. Figure S4 shows that the future temperature change is positively correlated with the current SD among the CMIP3 models over the Southern Ocean and a few other regions, but the correlation is weak at low-latitudes and other areas. This inter-model correlation depends on how the inter-model spreads were generated and it differs from the pattern correlation discussed above. One cannot always expect a positive inter-model correlation between the sizes of the current SD and future response.

Subtracting the ensemble mean patterns from the SD and future change maps from individual models reduces the spatial correlation (Figs S5 and S6), especially for the PW case. This is because the ensemble mean patterns capture most of the important distributions such as the land-sea contrast and the low-high latitude gradients. A model that happens to have its SD and future change patterns very close to the ensemble mean should contain small and noisy variations in its residual maps and thus likely shows low spatial correlations after the ensemble mean being subtracted. Clearly, this has little implication regarding the model’s performance. Likewise, a model with high pattern correlation in Fig. 2 does not imply it is a better model, since its SD patterns may or may not be realistic. Our results only suggest that models with more realistic patterns of variability for today’s climate are likely to predict more reliable change patterns for future climate.

## Additional Information

**How to cite this article**: Dai, A. Future Warming Patterns Linked to Today’s Climate Variability. *Sci. Rep.* **6**, 19110; doi: 10.1038/srep19110 (2016).

## Supplementary Material

Supplementary Information

## References

[b1] Räisänen. J. How reliable are climate models? Tellus A 59, 2–29 (2007).

[b2] Gleckler P. J., Taylor K. E. & Doutriaux. C. Performance metrics for climate models, J. Geophys. Res. 113, D06104, 10.1029/2007JD008972 (2008).

[b3] Macadam I., Pitman A. J., Whetton P. H. & Abramowitz. G. Ranking climate models by performance using actual values and anomalies: Implications for climate change impact assessments. Geophys. Res. Lett. 37, L16704, 10.1029/2010GL043877 (2010).

[b4] Knutti R., Furrer R., Tebaldi C., Cermak J. & Meehl. G. A. Challenges in combining projections from multiple climate models. J. Climate 23, 2739–2758 (2010).

[b5] Northrop P. J. & Chandler. R. E. Quantifying sources of uncertainty in projections of future climate. J. Climate 27, 8793–8808 (2014).

[b6] IPCC. Climate Change 2007: The Physical Science Basis. Contribution of Working Group I to the Fourth Assessment Report of the Intergovernmental Panel on Climate Change [Solomon S., Qin D., Manning M., Chen Z., Marquis M., Averyt K. B., Tignor M. & Miller H. L. (eds)]. Cambridge Univ. Press, Cambridge, UK and New York, NY, USA, 996 pp (2007).

[b7] IPCC. Climate Change 2013: The Physical Science Basis. Contribution of Working Group I to the Fifth Assessment Report of the Intergovernmental Panel on Climate Change [ Stocker T. F., Qin D., Plattner G.-K., Tignor M., Allen S. K., Boschung J., Nauels A., Xia Y., Bex V. & Midgley P. M. (eds)]. Cambridge Univ. Press, Cambridge, UK and New York, NY, USA, 1535 pp (2007).

[b8] Dai. A. Precipitation characteristics in eighteen coupled climate models. J. Climate 19, 4605–4630 (2006).

[b9] Monahan A. H., Fyfe J. C., Ambaum M. H. P., Stephenson D. B. & North. G. R. Empirical orthogonal functions: The medium is the message. J Climate 22, 6501–6514 (2009).

[b10] Meehl G. A., Covey C., Delworth T., Latif M., McAvaney B., Mitchell J. F. B., Stouffer R. J. & Taylor. K. E. The WCRP CMIP3 multimodel data set: A new era in climate change research. Bull. Am. Meteorol. Soc. 88, 1383–1394, 10.1175/BAMS-88-9-1383 (2007).

[b11] Taylor K. E., Stouffer R. J. & Meehl. G. A. An overview of CMIP5 and the experiment design. Bull. Amer. Meteorol. Soc. 93, 485–498 (2012).

[b12] Dai A., Fyfe J. C., Xie S.-P. & Dai. X. Decadal modulation of global surface temperature by internal climate variability. Nature Climate Change 5, 555–559, 10.1038/nclimate2605 (2015).

[b13] Brohan P., Kennedy J. J., Harris I., Tett S. F. B. & Jones. P. D. Uncertainty estimates in regional and global observed temperature changes: a new dataset from 1850. J. Geophysical Research 111, D12106, 10.1029/2005JD006548 (2006).

[b14] Rayner N. A., Brohan P., Parker D. E., Folland C. K., Kennedy J. J., Vanicek M., Ansell T. & Tett. S. F. B. Improved analyses of changes and uncertainties in marine temperature measured *in situ* since the mid-nineteenth century: the HadSST2 dataset. J. Climate 19, 446–469(2006).

[b15] Dai. A. Recent climatology, variability and trends in global surface humidity. J. Climate 19, 3589–3606 (2006).

[b16] Byrne M. P. & O’Gorman. P. A. Land–ocean warming contrast over a wide range of climates: Convective quasi-equilibrium theory and idealized simulations. J. Climate 26, 4000–4016 (2013).

[b17] Geoffroy O., Saint-Martin D. & Voldoire. A. Land-sea warming contrast: the role of the horizontal energy transport. Climate Dynamics 10.1007/s00382-015-2552-y (2015).

[b18] Lu J. & Cai. M. Quantifying contributions to polar warming amplification in an idealized coupled general circulation model. Clim. Dyn. 34, 669–687 (2009).

[b19] Stott P. A. & Jones. G. S. Variability of high latitude amplification of anthropogenic warming. Geophys Res Lett 36, L10701, 10710.11029/12009GL037698 (2009).

[b20] Screen J. A. & Simmonds. I. The central role of diminshing sea ice in recent Arctic temperature amplification. Nature 464, 1334–1337 (2010). 20428168 10.1038/nature09051

[b21] Bintanja1 R., Graversen1 R. G. & Hazeleger. Arctic winter warming amplified by the thermal inversion and consequent low infrared cooling to space. Nature Geosci. 4, 758–761 (2011).

[b22] Barsugli J. J., Shin S. I. & Sardeshmukh. P. D. Sensitivity of global warming to the pattern of tropical ocean warming. Clim Dynam 27, 483–492 (2006).

[b23] Xie S. P., Deser C., Vecchi G. A., Ma J., Teng H. Y. & Wittenberg. A. T. Global warming pattern formation: Sea surface temperature and rainfall. J Climate 23, 966–986 (2010).

[b24] Trenberth K. E., Fasullo J. & Smith. L. Trends and variability in column-integrated atmospheric water vapor. Clim Dynam 24, 741–758 (2005).

