# Supplementary material for: Future Warming Patterns Linked to Today’s Climate Variability
Source: Sci Rep. 2016 Jan 11;6:19110. doi: 10.1038/srep19110 (PMC4707535; doi:10.1038/srep19110)
Supplement: Supplementary Information [file srep19110-s1.doc]

**Supplementary Information**

Future Warming Patterns Linked to Today's Climate Variability

Aiguo Dai


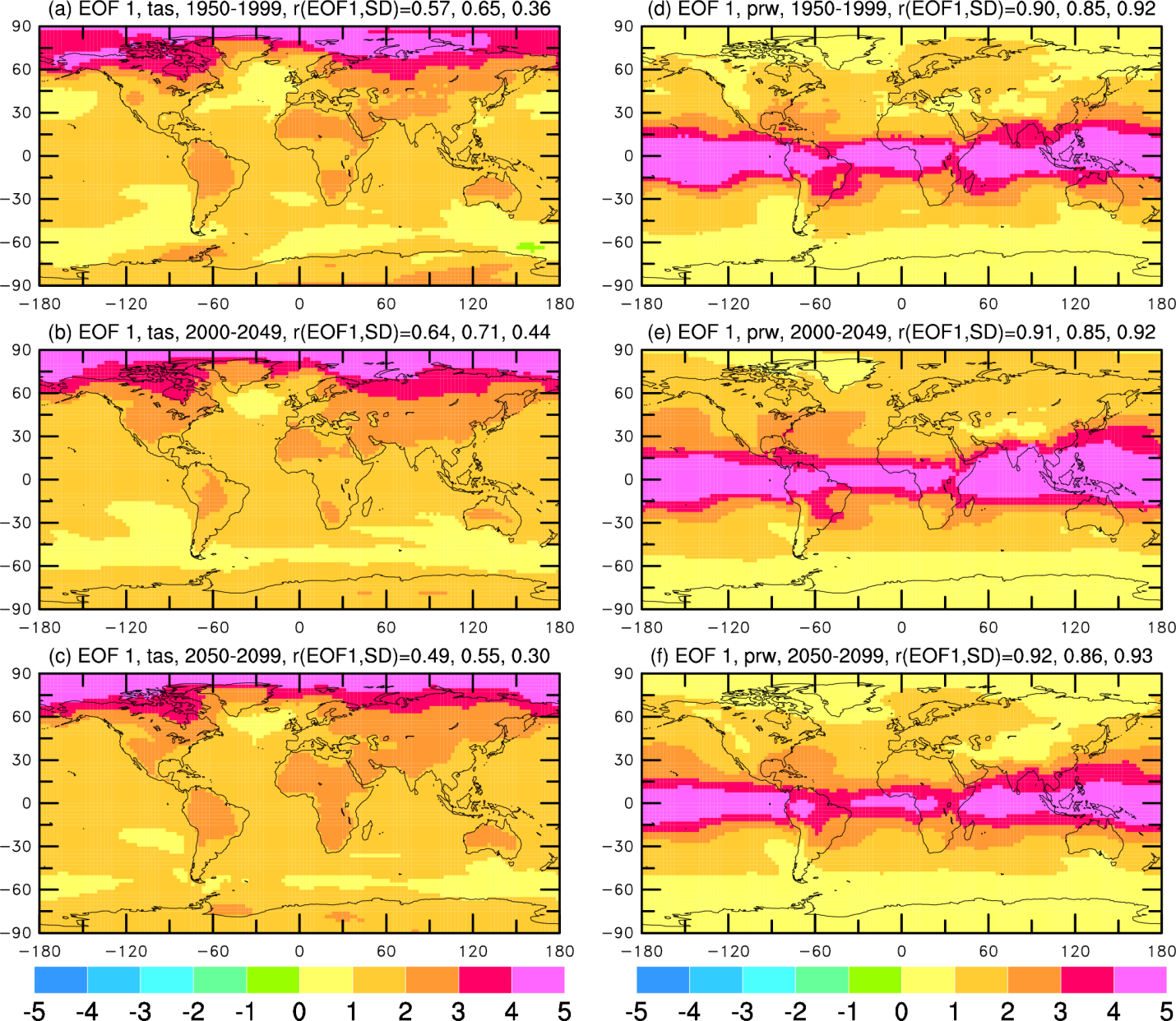


**FIG. S1.** **Left column**: The leading EOF of the monthly anomalies of surface air temperature simulated by 22 AR4 models during (**a**) 1950-1999, (**b**) 2000-2049, and (**c**) 2050-2099 under the A1B scenario. **Right column**: same as the left column but for total column water vapor content from 20 models. The area-weighted pattern correlation coefficient (*r*) with the standard deviation of 1950-1979 (cf. Fig. 1d,h) for the respective variable is shown on top of each panel for, from left to right, all areas, land and ocean only. All maps were created using NCAR Graphics library by the author.

**
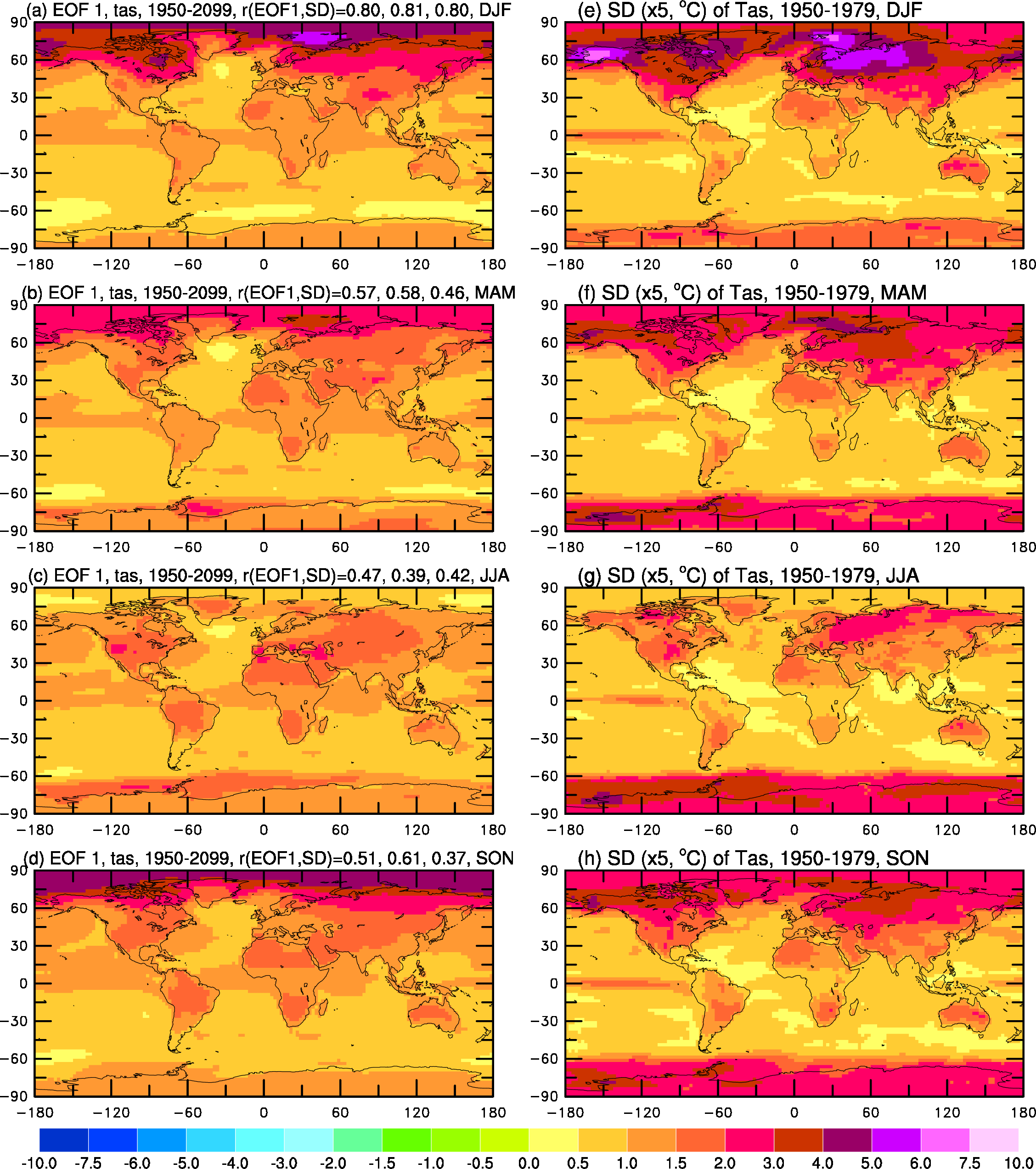
**

**FIG. S2.** Comparison between the spatial patterns of the EOF1 (left column) for 1950-2099 and SD (right column) for 1950-1979 of surface monthly temperatures within each season from 22 CMIP3 model simulations (multi-model ensemble averaged before the EOF analysis). The pattern correlation coefficient between the seasonal EOF1 and SD is shown on top of the panel in the left column for, from left to right, all areas, land and ocean only. All maps were created using NCAR Graphics library by the author.

**
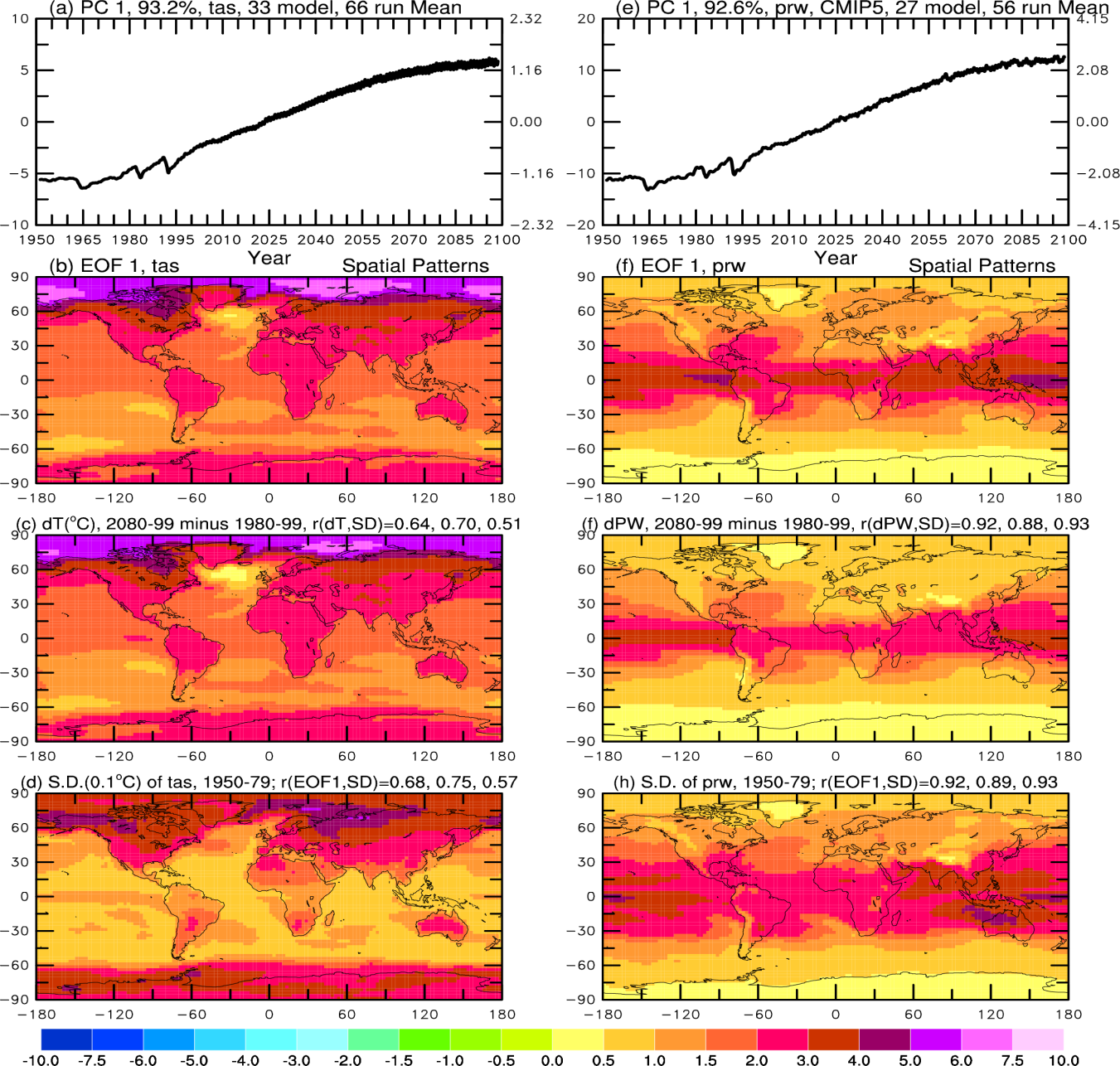
**

**FIG. S3. Left column:** The principal component (PC, **a,** left ordinate) and empirical orthogonal function (EOF, **b**) of the leading mode of the monthly anomalies (relative to the 1950-1979 mean annual cycle) of surface air temperature (SAT) from 33 CMIP5 models from their 20^th^ and 21^st^ (under the RCP4.5 scenario) century simulations. The EOF 1 pattern is compared with the patterns in (**c**) 2080-2099 minus 1980-1999 SAT differences (dT) and (**d**) the standard deviation (S.D.) of the monthly temperature anomalies during 1950-1979, with the pattern correlation coefficient (*r*) between the dT and SD (EOF1 and SD) is shown on top of panel **c (d)** for, from left to right, all areas, land and ocean only. In (**a**), the global-mean temperature anomalies (^o^C) associated with this EOF is shown on the right-side ordinate, and % variance explained by the mode is given on top of the panel. **Right column:** same as the left column but for precipitable water (PW) from 27 CMIP5 models and the unit is millimeters. To use the same color scale, values in (**f**) and (**h**) were multiplied by 0.4 and 6.0, respectively. The product of the PC and EOF coefficients yields the anomalies in one tenth of the given unit associated with the mode for a given time and location. All maps were created using NCAR Graphics library by the author.


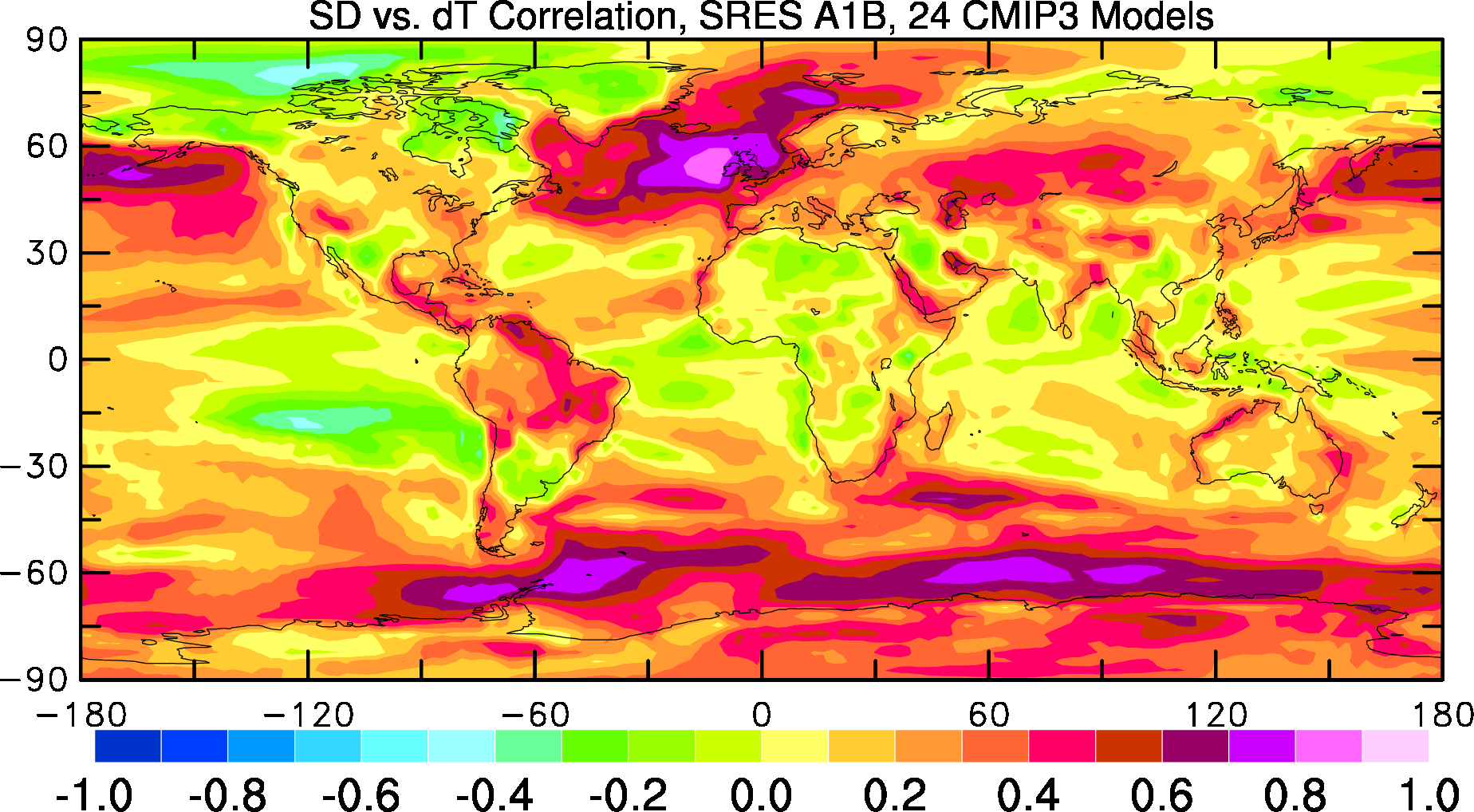


**FIG. S4.** Distributions of the inter-model correlation among 24 CMIP3 models between local S.D. of monthly surface air temperature (Tas) anomalies during 1950-1979 and local Tas change (dT) from 1980-1999 to 2080-2099 under the SRES A1B scenario. A positive (negative) correlation indicates that a model with a large S.D. tends to predict a large (small) dT. Correlations within ±0.4 are statistically insignificant at the 5% level.


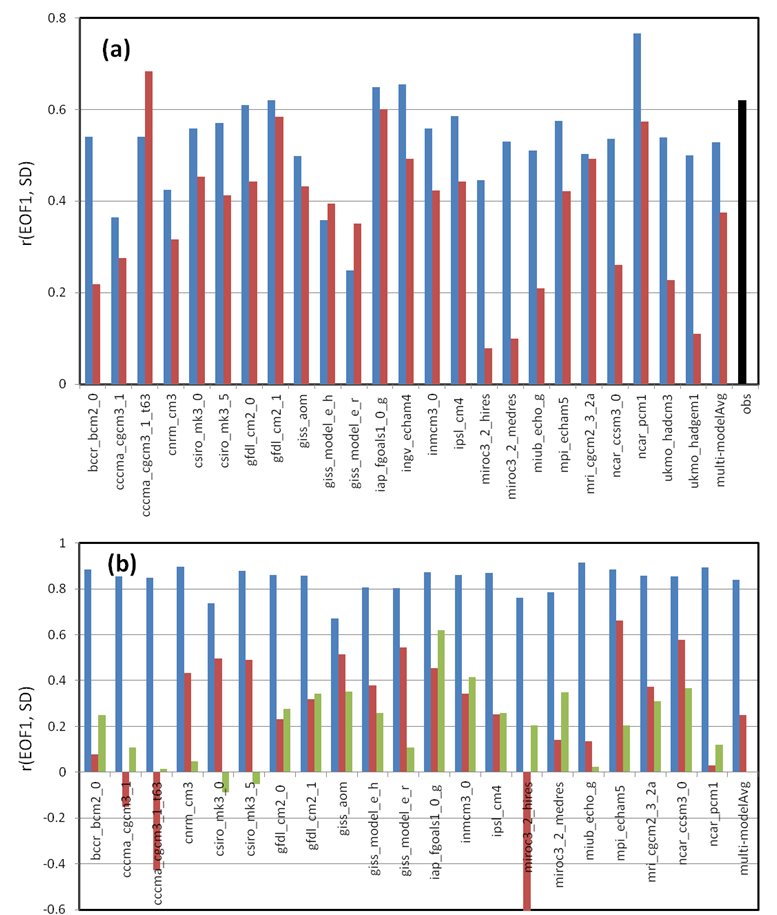


**FIG. S5**. The correlation coefficient (blue bars) between the spatial patterns of the standard deviation (SD) of the monthly anomalies during 1950-1979 and the leading EOF of the monthly anomalies during 1950-2099 under the A1B scenario simulated by individual CMIP3 coupled models and their average (Multi-ModelAvg) for (**a**) surface air temperature and (**b**) precipitable water. Also shown in (**a**) (black bar) is the case for observed surface monthly temperatures during 1950-2010. Also shown (dark red bar) is the correlation with the multi-model ensemble patterns were removed from both the SD and EOF1 maps. The green bar in (**b**) is the case in which the SD and EOF1 maps from the individual models were re-scaled to have the same global-mean values as those for the multi-model ensemble mean maps and then the ensemble mean maps were subtracted from the individual model maps before computing the spatial correlation. Thus, the red and green bars are still pattern correlations (cf. Fig. S6) but without the ensemble mean patterns in the SD and EOF maps.


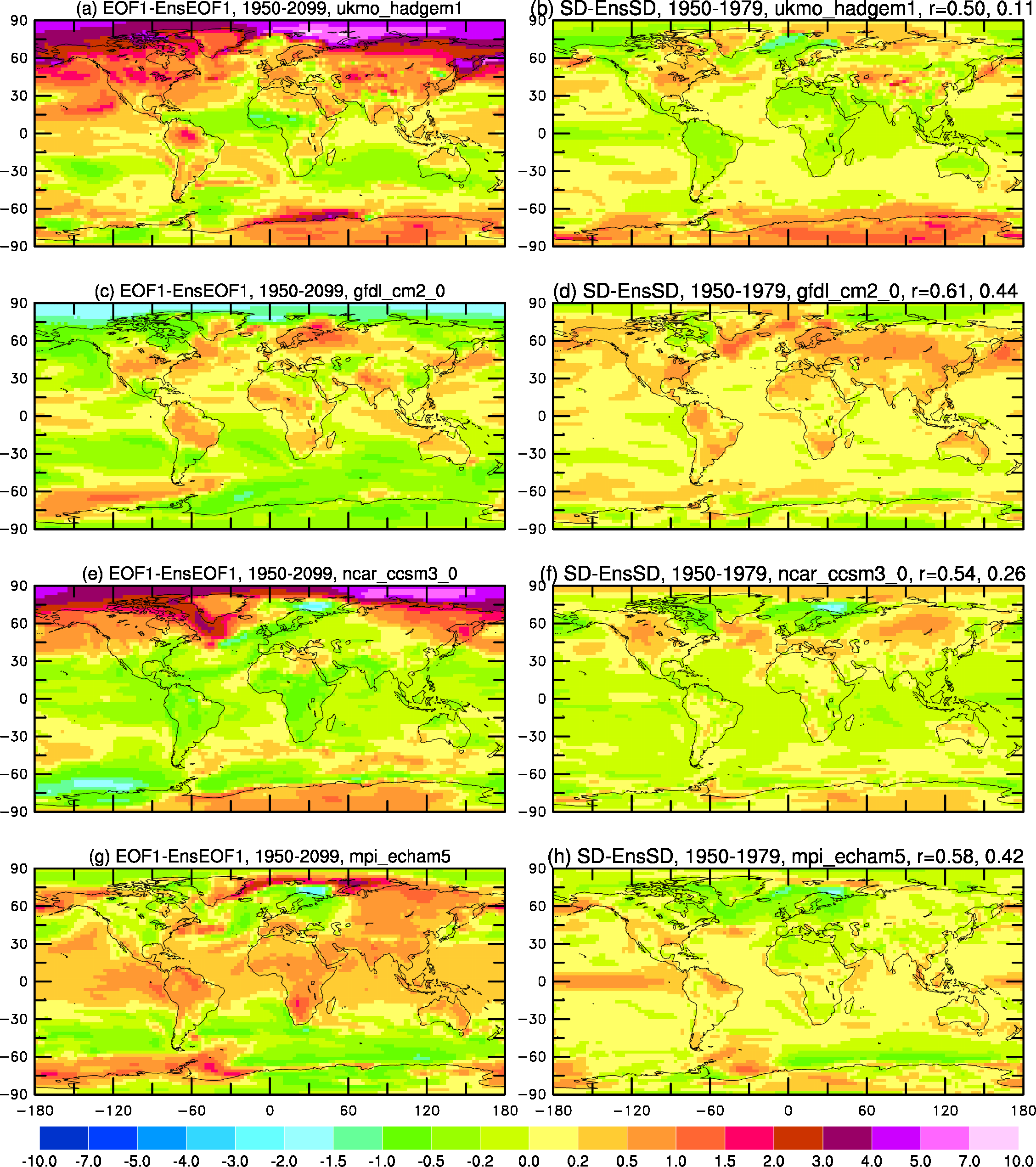


FIG. S6. Maps of the difference between a select model and the multi-model ensemble average for EOF 1 of 1950-2099 (left column) and the standard deviation (SD) of 1950-1979 of monthly surface air temperature. The spatial pattern correlation (*r*) between the EOF 1 and SD maps is shown on top of the right-side panels, from left to right, for the cases with and without the ensemble mean patterns.
